# User Authentication Using Inner-Wrist Skin Prints: Feasibility and Performance Assessment with Off-the-Shelf Fingerprint Sensor

**DOI:** 10.3390/s26041103

**Published:** 2026-02-08

**Authors:** Szymon Cygan, Patryk Lamprecht, Jakub Żmigrodzki, Jan Łusakowski-Milencki, Nikolaos Simopulos, Adrian Zarycki, Piotr Muranty

**Affiliations:** 1 Institute of Metrology and Biomedical Engineering, Warsaw University of Technology, 02-525 Warsaw, Poland; jakub.zmigrodzki@pw.edu.pl (J.Ż.); jan.lusakowski.dokt@pw.edu.pl (J.Ł.-M.); nikolaos.simopulos.stud@pw.edu.pl (N.S.); adrianzarycki@gmail.com (A.Z.); 2 Invis sp. z o. o., 00-682 Warsaw, Poland; plamprecht@inviswearables.com; 3 True Moves sp. z o. o., 03-733 Warszawa, Poland; pmuranty@inviswearables.com

**Keywords:** wrist biometrics, skin texture, wearable authentication, capacitive fingerprint sensor, biometric verification, wrist-worn devices

## Abstract

**Highlights:**

**What are the main findings?**

- Wrist skin print patterns can be verified using an off-the-shelf capacitive fingerprint sensor and an unmodified, closed fingerprint recognition algorithm.
- No false acceptances were observed in 86,897 impostor comparisons, establishing a conservative experimental upper bound on the false acceptance rate.

**What are the implications of the main findings?**

- Moderate wrist posture variation does not appear to be the dominant factor affecting verification performance under controlled acquisition conditions.
- The observed performance reflects the behavior of a fingerprint-oriented sensor and matcher applied to wrist skin texture, providing a baseline for future dedicated method development.

**Abstract:**

Wrist-worn devices enable new paradigms of implicit and continuous user authentication; however, identifying biometric modalities that combine reliability with practical integrability remains challenging. Inner-wrist skin texture represents a relatively unexplored biometric characteristic that may be acquired unobtrusively using commodity hardware. This study evaluates biometric verification based on inner-wrist skin texture using an off-the-shelf capacitive fingerprint sensor and an unmodified, manufacturer-provided fingerprint verification algorithm. Two experiments were conducted. Experiment 1 assessed baseline verification performance under controlled acquisition conditions in a cohort of 33 participants (21 male, 12 female; mean age 30.0 ± 16.9 years, range 10–71 years), yielding 1768 genuine authentication trials. Experiment 2 examined the effect of wrist posture variation under controlled flexion in a separate cohort of 15 participants (11 male, 4 female; mean age 30.9 years, range 18–49 years), with 3900 authentication trials recorded. Across 86,897 impostor comparisons in Experiment 1, no false acceptances were observed, corresponding to a conservative upper bound on the false acceptance rate of 6.7 × 10^−5^ at the 99.7% confidence level, while the false rejection rate was approximately 2.93%. In Experiment 2, the overall false rejection rate increased to 3.52%, with no clear monotonic relationship between wrist angle and verification performance within the tested range. The results demonstrate that inner-wrist skin texture can be captured and matched using fingerprint-oriented sensing and matching technology under controlled conditions, providing an experimental baseline for this biometric modality. At the same time, the use of a closed matching algorithm and a sensor designed for fingerprints limits interpretability and generalization. These findings motivate further investigation using dedicated recognition methods, larger sensing areas, and extended evaluation protocols tailored specifically to wrist skin print biometrics.

## 1. Introduction

Biometric authentication has become a central component of secure digital interactions, enabling reliable identity verification across consumer electronics, financial services, transportation systems, and regulated industrial environments. As digital ecosystems expand, regulatory frameworks increasingly require authentication methods that provide both robust security and low user friction. The forthcoming Payment Services Directive 3 (PSD3) and the accompanying Payment Services Regulation (PSR) introduce stricter requirements for Strong Customer Authentication (SCA), favoring biometric modalities that support continuous or passive verification, maintain resilience to spoofing, and ensure that sensitive biometric data remains processed on-device securely. These provisions encourage the development of wearable-integrated biometric systems that combine high security, transparency, and user convenience—particularly in contexts such as payments, physical access, and mobile identity services.

Traditional biometric technologies—such as fingerprints, facial recognition, and iris scanning—remain dominant in authentication systems, yet they exhibit persistent limitations under real-world conditions. Fingerprint recognition is highly sensitive to humidity, dryness, sensor aging, and dermatological conditions, which degrade data quality and lead to elevated error rates [1,2,3]. Spoofing attacks using synthetic fingerprints, 3D casts, or latent prints remain a major concern despite recent progress in presentation attack detection [4,5,6]. Another widely deployed modality is face recognition, which can be broadly divided into 2D and 3D approaches. Three-dimensional methods offer advantages such as improved robustness to illumination changes and cosmetics, as well as more accurate facial pose estimation [7]. Nevertheless, both 2D and 3D face recognition systems—as well as iris biometrics—are affected by occlusion, pose variability, and adverse acquisition conditions and may be vulnerable to high-quality presentation attacks, including masks or replay-based methods [8,9,10]. In practice, consumer biometric systems used in commercial devices typically do not disclose full false acceptance and false rejection characteristics; instead, security assurances are communicated through high-level vendor claims or compliance with certification frameworks rather than published performance curves. These systems frequently require explicit and precise user interaction—such as a stable pose, correct gaze direction, or consistent finger placement—which reduces throughput and limits usability in mobile or public environments [11,12]. Additionally, hygiene concerns associated with contact-based fingerprint scanners, highlighted during the COVID-19 pandemic, have increased demand for hygienic, seamless, and unobtrusive alternatives [13,14].

Driven by these limitations, research has increasingly turned toward wrist-based physiological and behavioral biometrics, which offer contactless acquisition, higher usability, improved integration with wearable devices, and naturally embedded liveness cues that enhance spoofing resistance. Ultrasonic impedance features used in the WristPass system enable secure continuous authentication with 96.7% accuracy [15]. Photoplethysmography-based “PressHeart” authentication reports 94.9% accuracy by exploiting individualized pressure-induced hemodynamic responses [16]. Soft wrist-worn arrays combining EMG and pressure sensing achieve gesture-recognition accuracies above 86% [17]. Other physiological traits at the wrist—such as subcutaneous vein patterns—can be captured using RGB or NIR imaging, with error rates frequently below 2% and as low as <0.1% when using specialized NIR hardware [18,19,20]. Work published so far has also demonstrated that the wrist exhibits rich surface ridge micro-features, including bifurcations, ridge endings, and bridges, which can be detected using deep learning architectures such as YOLOv11 and its derivatives, achieving detection accuracies above 94% [11,21,22]. Wrist-print biometrics have previously been explored using optical, image-based approaches; for example, Okafor and Longe [23] proposed a wrist skin print identification system employing palm-print–class optical acquisition hardware with full access to raw wrist images and classical DCT-based feature extraction. Complementary behavioral modalities arising from wrist motion dynamics, handwriting patterns, and electromyographic activity provide additional user-discriminative signatures. Wrist-motion and handwriting-based authentication approaches have shown strong feasibility [24], while EMG-based methods report stable multi-day performance with equal error rates around 3–4% [25,26,27]. Collectively, these emerging modalities expand the wrist into a versatile multimodal biometric site, offering a balance of accuracy, usability, and spoofing resistance that directly responds to—and in many cases alleviates—key limitations of traditional biometric systems.

Despite these advances, high-resolution inner-wrist skin micro-topography remains a largely unexplored modality. Anatomically, the inner wrist features stable ridge-like patterns analogous to fingerprints, yet its biometric potential has not been systematically evaluated using compact, off-the-shelf sensors. Leveraging this region would enable secure, intuitive authentication embedded directly into wrist-worn objects such as smartwatch straps, medical wearables, and watch clasps—without requiring explicit user action.

This study investigates the feasibility and accuracy of inner-wrist skin print-based biometric verification using a commercial, off-the-shelf capacitive fingerprint sensor. The primary objective is to determine whether standard, template-based biometric methods—implemented through a closed, manufacturer-provided verification algorithm—can reliably extract, represent, and match micro-texture patterns of the inner wrist under controlled and representative acquisition conditions. Using the BM-Lite Development Kit (Part number: 100018754; Fingerprint Cards AB, Gothenburg, Sweden) as a representative commercial platform, the study evaluates wrist skin print acquisition repeatability, sensitivity to sensor placement consistency, and overall matching performance under realistic handling scenarios. 

Two complementary experimental protocols were designed. Experiment 1 provides a controlled assessment of the intrinsic discriminative capability of inner-wrist skin prints using classical biometric performance metrics, including false acceptance rate (FAR) and false rejection rate (FRR). This experiment establishes a baseline performance reference for wrist skin print verification under stable acquisition conditions, enabling comparison with commonly reported benchmarks for biometric systems evaluated in laboratory settings. Experiment 2 examines the impact of wrist flexion and sensor repositioning on verification performance, specifically assessing whether controlled changes in wrist angle influence verification outcomes. By separating posture-related effects from acquisition variability, this experiment evaluates robustness to orientation changes that may occur during everyday use while avoiding claims of full biomechanical invariance.

The work presented here is technically aligned with the broader research direction outlined in the authors’ patent-pending system, WO 2025/177176—Wrist-Worn Device for Biometric User Identity Verification. However, the present study remains deliberately limited in scope to an experimental evaluation of inner-wrist skin prints as a standalone biometric modality, implemented using commercially available fingerprint sensing hardware and a closed verification algorithm. The reported results therefore assess practical feasibility rather than proposing or validating a deployable authentication system. By characterizing verification accuracy, error rates, and sensitivity to wrist repositioning, the study provides empirical evidence to inform future system-level developments, including multimodal extensions and robustness-enhancing strategies, without exceeding the experimental claims supported by the data.

## 2. Materials and Methods

### 2.1. Sensor Used

For the experimental evaluation of wrist skin print authentication, the BM-Lite Development Kit (Part number: 100018754; Fingerprint Cards AB, Sweden) was used. The BM-Lite is a compact, off-the-shelf biometric module intended for embedded authentication applications and integrates a capacitive fingerprint sensor, processing unit, and onboard template storage. The producer declares a sensor FRR of 1.4% and FAR of 1/500,000.

The sensor features an active area of 8 × 8 mm, a spatial resolution of 508 dpi, and captures 8-bit grayscale images suitable for high-resolution acquisition of fingerprint-like skin texture patterns. Although primarily designed for fingerprint acquisition, the module was used without hardware modification to capture wrist skin texture patterns.

**Figure 1:**
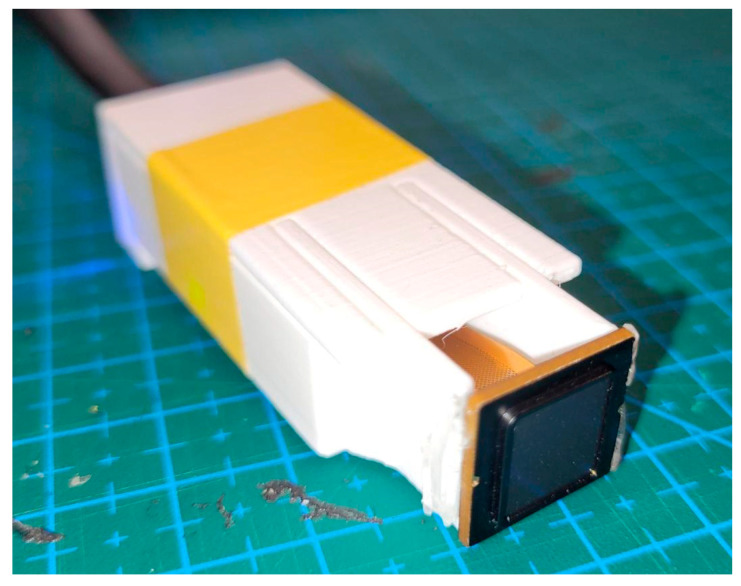
BM-Lite sensor embedded in the 3D-printed casing.

The sensor module was embedded in a custom 3D-printed casing designed for manual handling (Figure 1). The casing facilitated consistent sensor handling during data acquisition while allowing manual control of contact conditions. All signal processing, template extraction, and matching were performed locally on the BM-Lite module.

### 2.2. Enrollment Procedure, Operating Mode, and Template Handling

Enrollment and authentication in this study were performed using the standard operating modes provided by the BM-Lite firmware, without modification or custom algorithmic intervention. According to the manufacturer’s specification, enrollment is executed as a controlled multi-sample acquisition process, during which multiple captures of the biometric pattern are collected, internally assessed using a set of built-in image quality and consistency criteria, and then fused into a single reference template stored in the module’s non-volatile memory. In the present experiments, enrollment was performed once for each location on the participant’s skin, prior to testing, using the same acquisition procedure and quality criteria for all subjects and experiments.

All authentication trials were conducted using the fixed 1:1 verification mode supported by the BM-Lite module. This mode remained constant across all experiments, ensuring consistent decision logic, matching thresholds, and signal processing parameters throughout data collection. No adaptive mode switching or dynamic threshold adjustment was enabled during the study.

Importantly, as documented in the BM-Lite specification, templates stored in the module are static by default and are not updated during verification operations. Template updates or learning mechanisms can only be triggered explicitly through dedicated enrollment or maintenance commands issued by the host system. In this study, no such commands were used during authentication; consequently, the reference templates remained unchanged for the entire duration of the experiments.

This configuration guarantees that all verification results are based on comparisons against a fixed, pre-enrolled biometric reference, enabling full reproducibility of the experimental protocol and preventing uncontrolled performance drift due to incremental template adaptation.

### 2.3. Ethical Approval and Informed Consent

All experiments involving human subjects were conducted in accordance with ethical standards and were approved by the Research Ethics Committee at the Warsaw University of Technology (certificate no. 04/06/2025).

Prior to participation, all subjects were fully informed about the nature and purpose of the study, the procedures involved, potential risks, and their right to withdraw from the examination at any time without consequences. Written informed consent was obtained from all participants. For participants who were minors (three cases), written informed consent was obtained from their legal representatives.

### 2.4. Experiment 1: Controlled-Condition Wrist Skin Print Acquisition

#### 2.4.1. Data Acquisition Protocol

**Figure 2:**
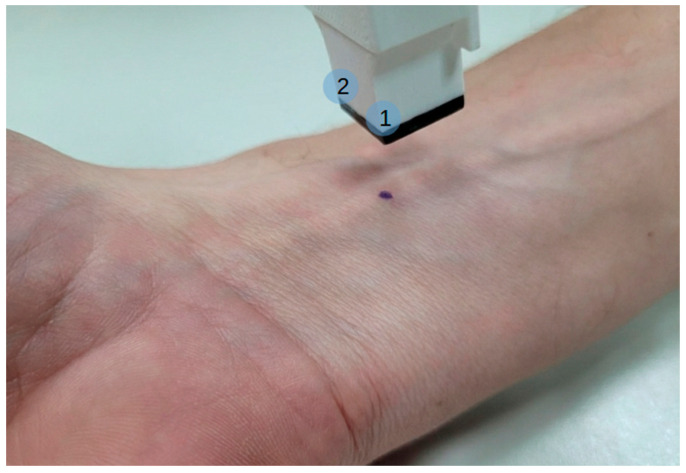
Sensor assembly over the wrist of a volunteer, with the pen mark for measurement site identification visible on the skin. Numbers 1 and 2 mark the corners that were aligned with the mark for two non-overlapping sites.

In the first experiment, participants were examined under controlled acquisition conditions with the hand and forearm placed on a flat surface (desk). To ensure reproducible localization of the measurement area across repeated acquisitions, a single pen mark (Figure 2) was applied to the volar side of the wrist at a distance of approximately 30 mm proximal to the distal wrist crease. This mark served exclusively as a visual reference for the operator. Two distinct, non-overlapping acquisition sites were defined proximally relative to this mark by controlled sensor placement: in the first site, the corner of the sensor indicated as position 1 in Figure 2 was aligned to touch the pen mark, while in the second site the sensor was shifted such that the corner indicated as position 2 touched the same mark. This procedure ensured a fixed spatial offset between the two sites while maintaining consistent orientation of the sensor with estimated placement accuracy of approximately ±1.5 mm. Both wrists were included in the protocol, resulting in skin prints acquired from two distinct locations on each wrist and a total of four measurement areas per participant.

Following enrollment, at least 10 verification attempts were conducted for each enrolled location, with a mean of 11.74 attempts, followed by additional 8 to 10 (mean 8.28) verification attempts per participant at other, unenrolled locations. All verification trials were performed manually by the experimenter using a handheld sensor assembly. Consequently, authentication performance reflects both the intrinsic properties of the wrist skin print modality and variability arising from manual sensor placement and skin–sensor contact conditions.

#### 2.4.2. Participants

A total of 33 participants took part in Experiment 1, including 21 male and 12 female subjects. The mean participant age was 30.0 ± 16.9 years, with a minimum age of 10 years and a maximum age of 71 years. In total, 1768 authentication trials were collected in Experiment 1.

### 2.5. Experiment 2: Wrist Flexion Study

#### 2.5.1. Mechanical Setup and Protocol

**Figure 3:**
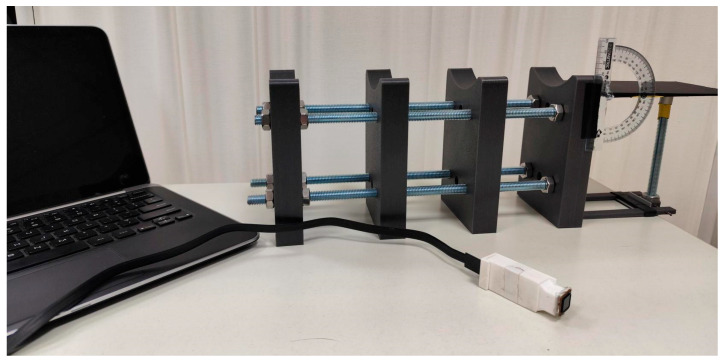
Hand supporting frame with pivoting platform for hand positioning.

**Figure 4:**
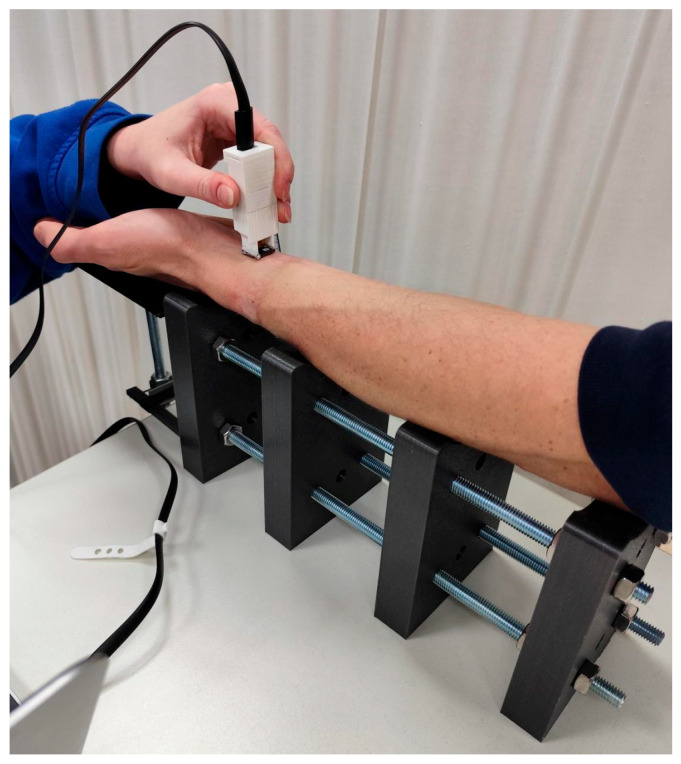
Hand of a volunteer during examination on the pivoting platform.

The second experiment was designed to evaluate the influence of wrist flexion on wrist skin print authentication performance. During testing, the participant’s forearm was positioned on a custom support assembly composed of four profiled 3D-printed blocks, which stabilized the forearm and minimized unintended motion (Figure 3). The hand rested on a pivoting platform, with the axis of rotation aligned with the anatomical wrist joint to ensure controlled flexion without translational displacement (Figure 4).

Wrist flexion was adjusted manually using a threaded-rod mechanism, enabling precise and repeatable positioning over an angular range from −30° to +30° relative to the horizontal plane, in 5° increments. Authentication performance was quantified using the false rejection rate (FRR) evaluated at each discrete wrist angle. Template enrollment was performed at the neutral wrist position (0°), and verification attempts acquired at each flexion angle were matched against the corresponding reference templates. To ensure consistent localization of the acquisition sites, the measurement areas were defined using the same pen-mark reference procedure as in Experiment 1. For each participant, two distinct, non-overlapping locations on the inner (volar) side of the left wrist were examined. Template enrollment for each location followed the same protocol as in Experiment 1. For each combination of wrist angle and measurement location, ten verification attempts were performed.

#### 2.5.2. Participants

A total of 15 volunteers participated in Experiment 2, including 11 male and 4 female subjects. The mean participant age was 30.9 years, with a minimum age of 18 years and a maximum age of 49 years. In total, 3900 authentication trials were recorded in Experiment 2.

### 2.6. Authentication and Matching Procedure

Each acquired image was compared internally against the full set of 50 enrolled templates stored on the device, comprising one template corresponding to the same subject and acquisition site (expected genuine comparison) and 49 templates associated with other subjects or different placements on the wrist, for which the acquired image constituted an impostor attempt.

The matching process yielded a binary verification decision—accept or reject—based on similarity thresholds defined internally within the BM-Lite firmware. These decision thresholds are fixed and not accessible to the user, reflecting the operation of a commercially realistic, ready-to-use biometric module rather than a configurable research prototype. During operation, no raw images, intermediate feature representations, or similarity scores were exposed to the host system.

All signal processing, template generation, storage, and matching operations were performed locally on the BM-Lite module. Throughout the experiments, biometric data remained confined to the device, with no external transmission or off-device processing. This configuration ensured that authentication outcomes reflected on-device performance and eliminated potential confounding effects related to external computation or data handling.

In Experiment 1, each verification attempt was evaluated against the enrolled template corresponding to the same wrist location, and the resulting outcomes were classified as true positives, false negatives, true negatives, or false positives depending on the origin of the presented wrist skin print. In Experiment 2, verification attempts acquired at varying wrist flexion angles were matched against templates enrolled at the neutral wrist position, and the resulting authentication outcomes were used to assess robustness to wrist repositioning.

### 2.7. Definition of Genuine and Impostor Comparisons (Experiment 1)

In Experiment 1, authentication outcomes were categorized as genuine or impostor comparisons based on the relationship between the verification attempt and the enrolled reference template. A genuine comparison was defined as a verification attempt originating from the same participant and the same enrolled measurement location as the reference template used for matching. Successful genuine comparisons were classified as true positives (TP), while unsuccessful genuine comparisons were classified as false negatives (FN).

An impostor comparison was defined as a verification attempt originating either from a different participant or from a wrist location that was not enrolled for the reference template being evaluated. This definition includes both inter-subject impostor attempts and intra-subject attempts involving unenrolled wrist locations. Impostor comparisons resulting in rejection were classified as true negatives (TN), whereas any acceptance of an impostor attempt was classified as a false positive (FP).

This classification framework enabled a clear separation between genuine and impostor presentations and provided the basis for computing false acceptance and false rejection rates under controlled acquisition conditions. During this study, a single sensor was used.

### 2.8. Performance Evaluation Metrics

System performance was evaluated using standard biometric verification metrics, specifically the false acceptance rate (FAR) and false rejection rate (FRR).

False acceptance rate (FAR) is defined as

(1) $$FAR=\frac{FP}{TN+FP}$$

False rejection rate (FRR) is defined as

(2) $$FRR=\frac{FN}{TP+FN}$$

These metrics were calculated directly from the authentication outcomes produced by the BM-Lite module for genuine and impostor comparisons in Experiment 1.

As the verification algorithm implemented in the BM-Lite module operates as a closed, ready-to-use system with internally fixed decision thresholds, it was not possible to adjust operating points or perform threshold sweeps. Consequently, estimation of the equal error rate (EER) was not feasible, and this metric is therefore not reported in the present study.

To quantify the false acceptance rate (FAR) in the absence of observed false acceptances, an exact one-sided binomial confidence bound was employed. Impostor authentication attempts were modeled as independent Bernoulli trials with the probability of success corresponding to a false acceptance event. For zero observed events (k = 0) across impostor comparisons, the upper confidence limit for the true FAR was computed using the exact Clopper–Pearson method, given by

(3) $${\mathrm{FAR}}_{\mathrm{upper}}=1-\alpha^{1/n}$$

where α denotes the significance level. This approach yields a conservative and statistically rigorous upper bound on the FAR and is commonly used in biometric performance evaluation when no false acceptances are observed [28].

In Experiment 2, performance evaluation was limited to analysis of the false rejection rate as a function of wrist flexion angle.

## 3. Results

### 3.1. Overview of Collected Data

Two experimental studies were conducted to evaluate the feasibility and robustness of wrist skin print-based authentication using an off-the-shelf biometric module.

In Experiment 1 (controlled conditions), data were collected from 33 participants, resulting in a total of 1768 authentication trials. Each trial generated one genuine comparison and multiple impostor comparisons, yielding a total of 86,897 impostor comparison events used for the estimation of false positive and true negative outcomes. This experiment was designed to quantify baseline authentication performance in terms of false acceptance rate (FAR) and false rejection rate (FRR) under manual acquisition conditions.

In Experiment 2 (wrist flexion study), data were collected from 15 participants, resulting in 3900 authentication trials. In this experiment, enrollment was performed at a neutral wrist position at the pen-marked positions, and subsequent verification attempts were acquired across a controlled range of wrist flexion angles. Performance evaluation in Experiment 2 focused exclusively on the analysis of false negative events as a function of wrist angle; impostor comparisons and false positive metrics were not considered.

The results of the two experiments are reported separately in the following sections, reflecting their distinct objectives and evaluation methodologies.

### 3.2. Authentication Performance Under Steady Conditions

#### 3.2.1. Overall False Acceptance and False Rejection Rates

Under controlled acquisition conditions, the wrist skin print authentication system demonstrated strong baseline performance. Across a total of 1768 authentication trials—including the additional 265 scans acquired from unenrolled wrist locations for all the participants—the system yielded 1478 true positive (TP) outcomes and 44 false negative (FN) events for genuine comparisons (Table 1). For impostor testing, 86,897 presentations were evaluated, all of which were correctly rejected, resulting in 86,897 true negatives (TN) and no false positives (FP = 0) (Table 1).

#### 3.2.2. Effect of Manual Sensor Placement

All wrist skin print acquisitions in Experiment 1 were performed manually by the operator using a handheld sensor assembly, introducing natural variability in sensor positioning, contact location, and applied pressure. This acquisition mode was intentionally selected to reflect realistic usage conditions rather than optimized laboratory alignment.

Given the relatively small number of observed false negative events (FN = 44), it is difficult to draw statistically robust conclusions regarding the specific influence of manual sensor placement on authentication performance. While variability in manual positioning can reasonably be assumed to contribute to some of the observed false negatives, the limited number of such events prevents reliable attribution of errors to individual acquisition factors. It should be noted, however, that sensor placement was supported by a visual aid in the form of a pen mark applied to the wrist, which served as a reference for consistent manual positioning across repeated acquisitions and was intended to improve placement repeatability.

Nevertheless, despite the absence of controlled positioning aids, the resulting false rejection rate of approximately 2.9% remains within a range generally considered acceptable for practical biometric authentication systems and is consistent with usability expectations associated with FIDO-compliant [29] biometric modalities. This indicates that wrist skin print authentication retains stable performance even under non-ideal, manually controlled acquisition conditions.

### 3.3. Authentication Performance Under Wrist Flexion

#### 3.3.1. Overall False Rejection Rates

In Experiment 2, authentication performance under wrist flexion was evaluated exclusively in terms of false negative events, with enrollment performed at a neutral wrist position and verification attempts acquired across a controlled range of wrist angles.

Aggregating all verification attempts across the examined wrist flexion range and measurement locations, the overall FRR was equal to 3.52%. This value reflects the cumulative effect of wrist posture variation on authentication reliability relative to the enrolled neutral-position templates.

#### 3.3.2. False Rejection Rate as a Function of Wrist Angle

**Figure 5:**
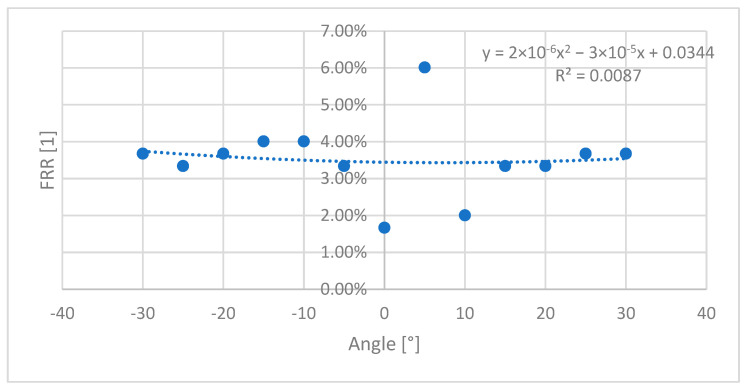
False rejection rate (FRR) values (blue circles) as a function of wrist flexion angle. Verification was performed at wrist angles ranging from −30° to +30° in 5° increments, with enrollment conducted at the neutral wrist position (0°). The dotted line represents a cubic polynomial fit to the data; the low coefficient of determination (R^2^ = 0.0087) indicates the absence of a clear correlation between FRR and wrist angle within the examined range.

Figure 5 shows the false rejection rate (FRR) as a function of wrist angle. The lowest FRR was observed at the neutral position (0°), with a value of 1.67%. At non-zero angles, FRR values ranged from 2.01% to 6.02%, with the maximum observed at +5°.

A quadratic polynomial fit applied to the FRR–angle relationship yielded a coefficient of determination of R^2^ = 0.0087, indicating that wrist angle accounts for less than 1% of the observed FRR variability. Accordingly, no systematic or monotonic dependence of FRR on wrist angle was identified within the examined range, aside from the expected performance improvement at the enrollment position.

#### 3.3.3. Qualitative Observations on the Scanning Procedure

In Experiment 2, enrollment was performed at the neutral wrist position (0°), and the corresponding verification scans were acquired without altering the position of the participant’s hand between enrollment and the initial verification attempts. This stationary transition between enrollment and scanning may have contributed to the lower false rejection rate observed at the neutral position.

For verification attempts acquired at non-zero wrist angles, the operator adjusted the position of the participant’s hand by modifying the wrist angle using the pivoting platform. Although the target skin region was visually marked to assist with repositioning, each adjustment required re-establishing contact between the sensor and the skin. Consequently, these scans were likely subject to increased variability in sensor placement, contact conditions, and local skin deformation compared to scans acquired without repositioning.

## 4. Discussion

The results presented in this study demonstrate that wrist skin print authentication, implemented using an off-the-shelf capacitive fingerprint sensor and a closed, manufacturer-defined verification algorithm, can achieve a favorable balance between security and practical usability under realistic acquisition conditions. In particular, the absence of false positives across a large number of impostor comparisons implies a very low likelihood of unauthorized acceptance, while the observed false rejection rates remain within ranges commonly regarded as acceptable for wearable biometric systems.

### 4.1. Security Performance in the Context of Non-Traditional Biometrics

The most notable outcome of Experiment 1 is the absence of false positives across 86,897 impostor comparisons, which permits a statistically justified upper bound of 6.7 × 10^−5^ to be placed on the false acceptance rate (FAR) at the 99.7% confidence level, based on the exact one-sided binomial upper bound [30]. This bound is substantially lower than the FAR requirements defined for FIDO BioLevel 1 (1:100) and BioLevel 2 (1:10,000) and approaches the more stringent FAR targets (e.g., 0.002% = 1:50,000) discussed in FIDO guidance for claims involving enhanced security assurances [29].

For reference, when used for its intended purpose of fingerprint verification, the BM-Lite sensor is specified by the manufacturer to achieve an FAR of approximately 1:500,000 with a corresponding false rejection rate (FRR) of 1.4% under standard operating conditions. Comparable off-the-shelf capacitive fingerprint sensors report FAR values in the range of 10^−4^–10^−6^ and FRR values between approximately 1% and 3%, depending on sensor size, resolution, and operating thresholds [21]. While these figures are reported for fingertip-based authentication and are not directly transferable to wrist skin print verification, they provide a relevant commercial benchmark for interpreting the observed impostor resistance achieved in the present study using the same hardware without algorithmic adaptation.

Notably, the security performance observed here exceeds FAR values reported for other non-traditional wrist-based biometric modalities, including photoplethysmography (PPG)-based authentication and dorsal hand vein recognition systems evaluated under laboratory or semi-controlled conditions [31,32,33,34]. Prior studies have shown that skin texture and micro-topography contain highly distinctive features that can support reliable user discrimination, even when acquired from body regions other than fingertips [35]. The present results extend these findings by demonstrating that such features can be captured from the volar wrist using commodity hardware, without algorithmic customization, while still achieving very strong resistance to impostor acceptance.

### 4.2. Usability and False Rejection Rates

The false rejection rates observed in this study (approximately 2.9% under steady conditions and 3.52% under wrist flexion) are consistent with FRR values reported for wrist-based wearable biometrics in the literature. Reviews and experimental studies of wrist-based PPG authentication commonly report FRR values in the range of 2–4% under controlled conditions, with higher values observed under motion or placement variability [31,32]. From this perspective, the FRR values reported here indicate usability comparable to other wearable biometric modalities, despite the use of a closed algorithm and manual acquisition.

It is important to note that the present study did not aim to optimize the trade-off between FAR and FRR through threshold tuning, as the verification algorithm operated with fixed, manufacturer-defined decision criteria. As a result, the reported FRR values should be interpreted as reflecting the behavior of a commercially realistic, fixed-threshold system rather than an optimally tuned research prototype. From a security perspective, the false acceptance rate (FAR) is the primary determinant of the system’s actual safety, particularly in fintech and payment-related applications where unauthorized access carries direct financial risk. In contrast, the false rejection rate (FRR) predominantly affects user experience, influencing perceived usability and convenience rather than core security guarantees.

### 4.3. Influence of Wrist Flexion and Acquisition Variability

Experiment 2 examined the influence of wrist flexion on authentication performance and found no measurable correlation between wrist angle and the false rejection rate (FRR) across the evaluated range. The very low coefficient of determination obtained from polynomial fitting (R^2^ = 0.0087) indicates that wrist angle accounts for only a negligible proportion of the observed FRR variability. This behavior contrasts with several biometric modalities whose performance degrades with changes in body part orientation, suggesting that inner-wrist skin texture is comparatively insensitive to variations in wrist angle.

Instead, the results point to acquisition consistency as the dominant factor influencing performance. This interpretation aligns with prior studies on wearable and contact-based biometrics, which identify sensor placement, contact pressure, and local repositioning as primary contributors to error rates [35,36]. In this study, the lowest FRR occurred at the enrollment angle, likely due to minimal repositioning between enrollment and verification, whereas measurements at other angles required replacement of the wrist on the sensor, introducing additional variability despite visual marking of the acquisition region. Similar findings have been reported for wrist-worn photoplethysmography (PPG) and motion-based biometric systems, where sensor displacement and motion-related artifacts outweigh posture-related effects [32].

Given the limited number of false negative events and the absence of a systematic relationship between FRR and wrist angle, the observed variations should be interpreted cautiously and cannot be attributed exclusively to wrist flexion.

### 4.4. Methodological Considerations and Limitations

As with many studies on non-traditional biometrics, the use of an off-the-shelf sensor and a closed verification algorithm introduces limitations in transparency and reproducibility. Previous surveys have emphasized that proprietary algorithms hinder direct cross-study comparison and complicate benchmarking against standardized evaluation frameworks [35,37]. While this constraint reflects realistic deployment scenarios, it also limits insight into feature-level behavior and error sources. In contrast, prior wrist-print studies based on optical, image-based acquisition—such as the work of Okafor and Longe [23]—operate on raw wrist images captured using palm-print–class sensors, enabling explicit region-of-interest selection, feature extraction, and algorithmic interpretability. The absence of such access in the present study restricts analysis to system-level performance metrics rather than feature-level characterization.

Additionally, the experiments were conducted within single sessions for each participant, and long-term stability of wrist skin prints across days or weeks was not evaluated. Longitudinal studies have been identified in the literature as a critical requirement for assessing the robustness and deployability of wearable biometric systems [36].

### 4.5. Implications and Future Work

Taken together, the results support the growing body of evidence that non-traditional wrist-based biometric modalities can achieve security and usability levels suitable for practical authentication applications. Wrist skin print authentication offers particular advantages in terms of unobtrusiveness and integration potential for wearable devices, complementing existing fingerprint-based solutions.

Future work should focus on (i) improving control and assessment of acquisition quality to reduce false negatives; (ii) evaluating longitudinal stability under real-world usage conditions; (iii) developing wrist-dedicated sensing and open processing pipelines that provide access to raw wrist skin data, enabling geometry-aware preprocessing and learning-based feature extraction, as demonstrated in optical wrist-print approaches [23]; and (iv) exploring multimodal fusion with complementary wrist-based signals, such as inertial or physiological data, as suggested by recent studies on multimodal wearable biometrics [38]. Such directions would help address current limitations and support more direct comparison with standardized biometric evaluation and certification frameworks.

## 5. Conclusions

This study provides an empirical feasibility assessment of wrist skin print-based biometric authentication using an off-the-shelf capacitive fingerprint sensor and an unmodified, manufacturer-provided verification algorithm originally designed for fingerprint recognition. Importantly, while the applied matching method was not adapted to the wrist, the acquired biometric pattern differs substantially from a conventional fingerprint, both in structure and in acquisition geometry. As such, the reported results should be interpreted as an evaluation of how a closed fingerprint recognition pipeline performs when applied to a distinctly different biometric trait.

Within the evaluated protocol and cohort, no false acceptances were observed across 86,897 impostor comparisons, corresponding to a conservative upper bound on the false acceptance rate of 6.7 × 10^−5^ at the 99.7% confidence level, while false rejection rates remained at approximately 3%. These findings indicate that wrist skin texture contains sufficient discriminative information to support reliable verification under controlled acquisition conditions, even when processed by a recognition method not optimized for this biometric characteristic.

Experiments involving controlled wrist flexion resulted in a modest increase in false rejection rate (3.52%), with the lowest error observed at the enrollment posture. No clear monotonic relationship between wrist angle and authentication performance was identified within the tested range, suggesting that moderate wrist repositioning alone is unlikely to be the dominant source of errors. Instead, acquisition-related factors such as contact consistency, pressure variation, and manual placement repeatability are likely contributors, although their individual effects were not explicitly quantified in this study.

At the same time, several limitations constrain broader interpretation of the results. The closed nature of the manufacturer-defined matching algorithm prevents access to raw images, feature representations, or similarity scores, limiting interpretability, reproducibility, and direct comparison with alternative biometric approaches. Moreover, the fingerprint sensor used in this work was dimensioned for fingertip acquisition. In contrast, wrist skin prints can be captured from a substantially larger anatomical area, potentially providing richer and more distinctive biometric information than is available to the present setup.

Consequently, developing a dedicated wrist skin print recognition method—designed explicitly for the geometry, scale, and texture characteristics of the wrist—appears both justified and worthwhile. The results reported here provide an initial indication of the level of performance that may be achievable with such a method, while retaining all limitations associated with the experimental design, population size, and acquisition protocol.

Future work should therefore focus on dedicated algorithm development, larger-area sensing, long-time stability analysis, and evaluation across larger and more diverse populations. Such studies are necessary to determine whether wrist skin print biometrics can offer improved reliability, robustness, and security properties beyond those observed using repurposed fingerprint technology. Overall, this work establishes an experimental baseline and motivation for further research, rather than a definitive validation of wrist skin print biometrics for security-critical or large-scale deployment scenarios.

## 6. Patents

The concepts investigated in this study are related to a patent-pending wrist-worn biometric identity verification system and method, which covers the use of wrist skin texture as a biometric modality in combination with wearable device architectures. The experimental results reported in this manuscript provide empirical validation of selected assumptions underlying this invention and support its applicability to practical wrist-worn authentication systems (WO 2025/177176).

## Figures and Tables

**Table 1:** Confusion matrix summary for Experiment 1.

Outcome Type	Count
True Positives (TP)	1478
False Negatives (FN)	44
True Negatives (TN)	86,897
False Positives (FP)	0
Falce Acceptance Rate (FAR)	0.0%
False Rejection Rate (FRR)	2.93%

## Data Availability

The data presented in this study are available on request from the corresponding author due to ethical and privacy restrictions associated with sensitive biometric data.

## Abbreviations

The following abbreviations are used in this manuscript:

TP	True Positive
TN	True Negative
FP	False Positive
FN	False Negative
FAR	False Acceptance Rate
FRR	False Rejection Rate
Dpi	Dots per inch
IRB	Institutional Review Board
FIDO	Fast Identity Online
